# *N*′-(3-Thienylmethyl­ene)pyridine-2-carbohydrazide

**DOI:** 10.1107/S1600536808004960

**Published:** 2008-03-14

**Authors:** Haojin Cheng, Brandon Djukic, Laura E. Harrington, James F. Britten, Martin T. Lemaire

**Affiliations:** a Department of Chemistry, Brock University, 500 Glenridge Avenue, St Catharines, Ontario, Canada L2S 3A1; b McMaster University, Department of Chemistry, 1280 Main Street W., Hamilton, Ontario, Canada L8S 4M1

## Abstract

The title compound, C_11_H_9_N_3_OS, was prepared to investigate the coordination chemistry of thio­phene-containing ligands as precursors to inter­esting metallopolymers. The mol­ecule is nearly planar. The angle between the thio­phene and pyridine rings is 8.63 (4)° and features the expected *trans* configuration about the imine bond. The structure is stabilized by a weak inter­molecular N—H⋯O hydrogen bond. The distance between centroids of adjacent thio­phene rings [3.67 (8) Å] suggests the presence of π–π inter­actions.

## Related literature

The preparation and coordination chemistry of a similar compound containing a 2-substituted thio­phene were reported previously by El-Motaleb *et al*. (2005 ▶); however, no structural details were provided. For related literature and structures of other mol­ecules containing the pyridine-2-carbonohydrazide system, see: Klingele & Brooker (2004 ▶); Xie *et al.* (2006 ▶); Zhang *et al.* (2006 ▶).
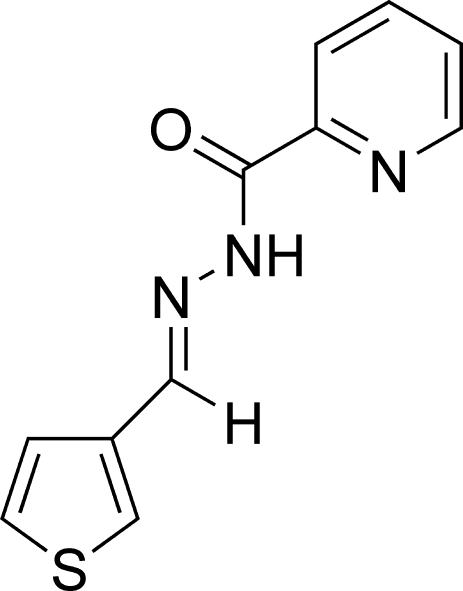


## Experimental

### 

#### Crystal data

C_11_H_9_N_3_OS
*M* *_r_* = 231.27
Monoclinic, 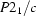

*a* = 11.6817 (3) Å
*b* = 9.1454 (3) Å
*c* = 10.0890 (3) Å
β = 103.230 (1)°
*V* = 1049.24 (5) Å^3^
*Z* = 4
Mo *K*α radiation
μ = 0.29 mm^−1^
*T* = 173 (2) K
0.40 × 0.30 × 0.20 mm

#### Data collection

Bruker APEXII CCD area-detector diffractometer
Absorption correction: multi-scan (*SADABS*; Sheldrick, 1996 ▶) *T*_min_ = 0.908, *T*_max_ = 0.959
32428 measured reflections
6449 independent reflections
5355 reflections with *I* > 2σ(*I*)
*R*_int_ = 0.024

#### Refinement

*R*[*F* ^2^ > 2σ(*F* ^2^)] = 0.035
*wR*(*F* ^2^) = 0.109
*S* = 1.05
6449 reflections
181 parameters
H atoms treated by a mixture of independent and constrained refinement
Δρ_max_ = 0.48 e Å^−3^
Δρ_min_ = −0.44 e Å^−3^

### 

Data collection: *APEX2* (Bruker, 2006 ▶); cell refinement: *APEX2*; data reduction: *APEX2*; program(s) used to solve structure: *SHELXTL* (Sheldrick, 2008 ▶); program(s) used to refine structure: *SHELXTL*; molecular graphics: *ORTEP-3 for Windows* (Farrugia, 1997 ▶); software used to prepare material for publication: *SHELXTL*.

## Supplementary Material

Crystal structure: contains datablocks I, global. DOI: 10.1107/S1600536808004960/fl2185sup1.cif

Structure factors: contains datablocks I. DOI: 10.1107/S1600536808004960/fl2185Isup2.hkl

Additional supplementary materials:  crystallographic information; 3D view; checkCIF report

## Figures and Tables

**Table 1:** Hydrogen-bond geometry (Å, °)

*D*—H⋯*A*	*D*—H	H⋯*A*	*D*⋯*A*	*D*—H⋯*A*
N2—H2⋯O1^i^	0.83 (1)	2.38 (1)	3.0717 (8)	140 (1)

Symmetry code: (i) 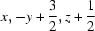.

**Fig. 1.**
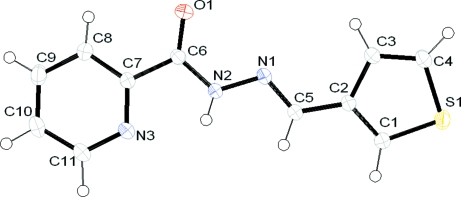
The molecular structure of the title compound with atom labels and 50% probability displacement ellipsoids for non-H atoms.

**Fig. 2.**
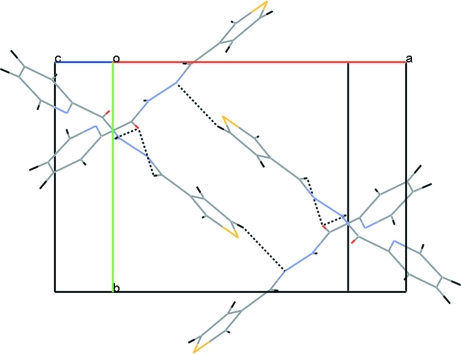
The packing of the title compound viewed along the c axis. The intermolecular hydrogen bond (N2—H2···O1) and other close contacts (H4···N1 and H5···O1) are indicated as dotted lines.

## supplementary crystallographic information

### Comment

We are interested in the coordination chemistry of thiophene containing ligands as precursors to interesting metallopolymers. The title compound (I, Fig. 1) features a pyridine-2-carbonohydrazide moiety grafted onto the 3 position of the thiophene ring and offers a number of possible coordination modes to metal ions, which we are currently exploring.

Bond lengths and angles are in the normal range reported for other molecules containing the pyridine-2-carbonohydrazide moiety (Xie *et al.*, 2006; Zhang *et al.*, 2006). Bond parameters within the thiophene and pyridine rings are also within normal ranges. The C5 - N1 bond is 1.284 (8) Å, typical for a double bond and features the expected *trans* configuration. The C6 - N2 bond of 1.356 (8) Å is in the range between the expected values for purely single or double bonds as a result of the π-conjugation. The molecule is nearly planar; the angle between the thiophene and pyridine ring is 8.63° (4). The structure is stabilized by an intermolecular hydrogen-bond (H2···O1 = 2.38 (1) Å) between the amide hydrogen and carbonyl oxygen atoms. Other weak intermolecular interactions are suggested by close contacts between H4···N1 (2.591 (14) Å) and H5···O1 (2.609 (12) Å), Fig. 2. The structure is further stabilized by π-π interactions between adjacent thiophene rings (ring centroids are 3.6720(0.0818 Å apart).

### Experimental

Pyridine-2-carbonohydrazide (Klingele & Brooker, 2004) (2.28 g, 16.6 mmol)was dissolved in 50 ml of absolute ethanol and cooled in an ice-water bath. A solution of 3-formylthiophene (2.91 g, 17.0 mmol) in 25 ml of absolute ethanol was added slowly dropwise to the cold hydrazide solution. Following the addition the ice-water bath was removed and the reaction was let stir at room temperature for 4 hr. While warming to room temperature, the appearance of a white microcrystalline precipitate was observed. The reaction flask was cooled in ice and the product was isolated by vacuum filtration, washed with cold ethanol and dried (yield 2.5 g, 65%). The compound was recrystallized by slow evaporation of a methanol solution to give large transparent blocks. MS (EI) = m/*z* 231 (*M*^+^, 20%), 79 (py^+^, 100%). FT—IR (KBr pellet) = 3295 (w, νN-H), 3072 (w), 1677 (s, νC=O), 1607 (*m*), 1533 (*s*), 1344 (*m*), 799 (*m*), 741 (*m*), 603 cm^-1^ (*m*). ^1^H NMR (CDCl_3_) = δ 10.91 (s, 1H, N—H), 8.60 (d, 1H, Ar—H), 8.42 (s, 1H, H—C=N), 8.33 (d, 1H, Ar—H), 7.92 (dd, 1H, Ar—H), 7.70 (d, 1H, Ar—H), 7.66 (d, 1H, Ar—H), 7.5 (dd, 1H, Ar—H), 7.38 (dd, 1H, Ar—H).

### Refinement

All non-hydrogen atoms were refined using anisotropic thermal parameters and hydrogen atoms were determined using the difference map and refined using isotropic thermal parameters.

### Crystal data

**Table:**

C_11_H_9_N_3_OS	*F*_000_ = 480
*M**_r_* = 231.27	*D*_x_ = 1.464 Mg m^−^^3^
Monoclinic, *P*2(1)/*c*	Mo *K*α radiation λ = 0.71073 Å
Hall symbol: -P 2ybc	Cell parameters from 9922 reflections
*a* = 11.6817 (3) Å	θ = 2.9–39.5º
*b* = 9.1454 (3) Å	µ = 0.29 mm^−^^1^
*c* = 10.0890 (3) Å	*T* = 173 (2) K
β = 103.2300 (10)º	Block, colourless
*V* = 1049.24 (5) Å^3^	0.40 × 0.30 × 0.20 mm
*Z* = 4	

### Data collection

**Table:**

Bruker APEXII CCD area-detector diffractometer	6449 independent reflections
Radiation source: fine-focus sealed tube	5355 reflections with *I* > 2σ(*I*)
Monochromator: graphite	*R*_int_ = 0.024
*T* = 173(2) K	θ_max_ = 40.5º
φ and ω scans	θ_min_ = 1.8º
Absorption correction: multi-scan(SADABS; Sheldrick, 1996)	*h* = −21→20
*T*_min_ = 0.908, *T*_max_ = 0.959	*k* = −16→16
32428 measured reflections	*l* = −18→14

### Refinement

**Table:**

Refinement on *F*^2^	Secondary atom site location: difference Fourier map
Least-squares matrix: full	Hydrogen site location: inferred from neighbouring sites
*R*[*F*^2^ > 2σ(*F*^2^)] = 0.035	H atoms treated by a mixture of independent and constrained refinement
*wR*(*F*^2^) = 0.109	*w* = 1/[σ^2^(*F*_o_^2^) + (0.0599*P*)^2^ + 0.1403*P*] where *P* = (*F*_o_^2^ + 2*F*_c_^2^)/3
*S* = 1.05	(Δ/σ)_max_ = 0.001
6449 reflections	Δρ_max_ = 0.48 e Å^−^^3^
181 parameters	Δρ_min_ = −0.44 e Å^−^^3^
Primary atom site location: structure-invariant direct methods	Extinction correction: none

### Special details

**Table:**

Geometry. All e.s.d.'s (except the e.s.d. in the dihedral angle between two l.s. planes) are estimated using the full covariance matrix. The cell e.s.d.'s are taken into account individually in the estimation of e.s.d.'s in distances, angles and torsion angles; correlations between e.s.d.'s in cell parameters are only used when they are defined by crystal symmetry. An approximate (isotropic) treatment of cell e.s.d.'s is used for estimating e.s.d.'s involving l.s. planes.
Refinement. Refinement of *F*^2^ against ALL reflections. The weighted *R*-factor *wR* and goodness of fit *S* are based on *F*^2^, conventional *R*-factors *R* are based on *F*, with *F* set to zero for negative *F*^2^. The threshold expression of *F*^2^ > σ(*F*^2^) is used only for calculating *R*-factors(gt) *etc*. and is not relevant to the choice of reflections for refinement. *R*-factors based on *F*^2^ are statistically about twice as large as those based on *F*, and *R*- factors based on ALL data will be even larger.

### Fractional atomic coordinates and isotropic or equivalent isotropic displacement parameters (Å^2^)

**Table:**

	*x*	*y*	*z*	*U*_iso_*/*U*_eq_	
S1	0.463771 (16)	0.23464 (2)	0.454041 (19)	0.02568 (5)	
O1	0.85993 (5)	0.78873 (6)	0.23484 (5)	0.02601 (10)	
C1	0.58685 (6)	0.33166 (8)	0.52713 (7)	0.02334 (11)	
H1	0.6218 (12)	0.3226 (15)	0.6255 (14)	0.038 (3)*	
N1	0.76136 (5)	0.59050 (6)	0.38381 (5)	0.01975 (9)	
N2	0.86362 (5)	0.66685 (7)	0.43407 (6)	0.02150 (10)	
H2	0.8983 (11)	0.6677 (15)	0.5159 (14)	0.041 (3)*	
C2	0.62431 (5)	0.41837 (7)	0.43437 (6)	0.01909 (9)	
C3	0.54986 (6)	0.40473 (8)	0.30101 (6)	0.02346 (11)	
H3	0.5598 (11)	0.4576 (15)	0.2219 (14)	0.038 (3)*	
N3	1.06794 (5)	0.77731 (7)	0.55311 (6)	0.02522 (11)	
C4	0.45955 (6)	0.30881 (8)	0.29705 (7)	0.02585 (12)	
H4	0.4001 (12)	0.2786 (15)	0.2207 (14)	0.039 (3)*	
C5	0.72865 (5)	0.50925 (7)	0.47217 (6)	0.02064 (10)	
H5	0.7741 (11)	0.5050 (14)	0.5620 (12)	0.032 (3)*	
C6	0.90780 (5)	0.75931 (7)	0.35325 (6)	0.01910 (10)	
C7	1.02436 (5)	0.82307 (7)	0.42481 (6)	0.01914 (10)	
C8	1.08096 (6)	0.92324 (8)	0.35862 (7)	0.02498 (12)	
H8	1.0462 (10)	0.9482 (14)	0.2685 (12)	0.030 (3)*	
C9	1.18816 (7)	0.98075 (9)	0.42927 (9)	0.02855 (13)	
H9	1.2275 (12)	1.0512 (16)	0.3916 (13)	0.040 (3)*	
C10	1.23335 (6)	0.93561 (9)	0.56184 (9)	0.02901 (13)	
H10	1.3068 (12)	0.9703 (17)	0.6115 (13)	0.042 (4)*	
C11	1.17048 (6)	0.83398 (10)	0.61907 (8)	0.02998 (14)	
H11	1.2008 (13)	0.7924 (18)	0.7155 (16)	0.050 (4)*	

### Atomic displacement parameters (Å^2^)

**Table:**

	*U*^11^	*U*^22^	*U*^33^	*U*^12^	*U*^13^	*U*^23^
S1	0.02498 (8)	0.02603 (8)	0.02706 (9)	−0.00757 (5)	0.00809 (6)	−0.00236 (5)
O1	0.0262 (2)	0.0318 (2)	0.01804 (19)	−0.00370 (18)	0.00077 (16)	0.00251 (17)
C1	0.0225 (2)	0.0263 (3)	0.0206 (2)	−0.0040 (2)	0.00363 (19)	0.0013 (2)
N1	0.01699 (19)	0.0214 (2)	0.0198 (2)	−0.00233 (16)	0.00210 (15)	−0.00098 (16)
N2	0.0187 (2)	0.0259 (2)	0.0182 (2)	−0.00545 (17)	0.00069 (16)	0.00143 (17)
C2	0.0182 (2)	0.0192 (2)	0.0193 (2)	−0.00147 (17)	0.00317 (17)	−0.00077 (17)
C3	0.0258 (3)	0.0244 (3)	0.0189 (2)	−0.0053 (2)	0.00249 (19)	−0.00152 (19)
N3	0.0194 (2)	0.0311 (3)	0.0230 (2)	−0.00334 (19)	0.00019 (17)	0.0037 (2)
C4	0.0265 (3)	0.0271 (3)	0.0223 (3)	−0.0072 (2)	0.0022 (2)	−0.0045 (2)
C5	0.0191 (2)	0.0224 (2)	0.0193 (2)	−0.00278 (18)	0.00200 (17)	−0.00012 (18)
C6	0.0181 (2)	0.0202 (2)	0.0184 (2)	−0.00070 (17)	0.00300 (17)	−0.00088 (17)
C7	0.0173 (2)	0.0199 (2)	0.0199 (2)	−0.00059 (17)	0.00357 (17)	−0.00080 (17)
C8	0.0246 (3)	0.0252 (3)	0.0248 (3)	−0.0050 (2)	0.0050 (2)	0.0016 (2)
C9	0.0240 (3)	0.0273 (3)	0.0346 (3)	−0.0067 (2)	0.0072 (2)	0.0005 (2)
C10	0.0191 (2)	0.0302 (3)	0.0355 (3)	−0.0043 (2)	0.0017 (2)	−0.0033 (3)
C11	0.0210 (3)	0.0380 (4)	0.0271 (3)	−0.0042 (2)	−0.0023 (2)	0.0025 (3)

### Geometric parameters (Å, °)

**Table:**

S1—C1	1.7067 (7)	N3—C11	1.3347 (9)
S1—C4	1.7133 (8)	N3—C7	1.3446 (9)
O1—C6	1.2274 (8)	C4—H4	0.953 (14)
C1—C2	1.3724 (9)	C5—H5	0.941 (12)
C1—H1	0.987 (13)	C6—C7	1.5052 (8)
N1—C5	1.2841 (8)	C7—C8	1.3873 (9)
N1—N2	1.3764 (7)	C8—C9	1.3944 (10)
N2—C6	1.3559 (8)	C8—H8	0.935 (12)
N2—H2	0.832 (14)	C9—C10	1.3838 (12)
C2—C3	1.4306 (9)	C9—H9	0.922 (14)
C2—C5	1.4523 (8)	C10—C11	1.3895 (11)
C3—C4	1.3655 (10)	C10—H10	0.944 (14)
C3—H3	0.963 (14)	C11—H11	1.029 (15)
			
H4···N1^i^	2.591 (14)	H5···O1^ii^	2.609 (12)
			
C1—S1—C4	92.11 (3)	C2—C5—H5	119.0 (8)
C2—C1—S1	111.98 (5)	O1—C6—N2	124.80 (6)
C2—C1—H1	127.7 (8)	O1—C6—C7	122.86 (6)
S1—C1—H1	120.3 (8)	N2—C6—C7	112.34 (5)
C5—N1—N2	114.13 (5)	N3—C7—C8	123.49 (6)
C6—N2—N1	120.83 (5)	N3—C7—C6	116.28 (5)
C6—N2—H2	115.1 (9)	C8—C7—C6	120.23 (6)
N1—N2—H2	123.7 (9)	C7—C8—C9	118.31 (7)
C1—C2—C3	111.77 (6)	C7—C8—H8	118.7 (7)
C1—C2—C5	122.07 (6)	C9—C8—H8	123.0 (7)
C3—C2—C5	126.15 (6)	C10—C9—C8	118.74 (7)
C4—C3—C2	112.51 (6)	C10—C9—H9	119.4 (8)
C4—C3—H3	122.5 (8)	C8—C9—H9	121.8 (8)
C2—C3—H3	124.9 (8)	C9—C10—C11	118.70 (7)
C11—N3—C7	117.26 (6)	C9—C10—H10	120.8 (8)
C3—C4—S1	111.64 (5)	C11—C10—H10	120.5 (8)
C3—C4—H4	128.8 (8)	N3—C11—C10	123.50 (7)
S1—C4—H4	119.6 (8)	N3—C11—H11	113.8 (9)
N1—C5—C2	120.94 (6)	C10—C11—H11	122.7 (8)
N1—C5—H5	120.0 (8)		
			
C4—S1—C1—C2	−0.15 (6)	C11—N3—C7—C8	0.86 (11)
C5—N1—N2—C6	−178.74 (6)	C11—N3—C7—C6	−179.13 (7)
S1—C1—C2—C3	0.21 (8)	O1—C6—C7—N3	−177.94 (7)
S1—C1—C2—C5	−178.92 (5)	N2—C6—C7—N3	1.78 (8)
C1—C2—C3—C4	−0.17 (9)	O1—C6—C7—C8	2.06 (10)
C5—C2—C3—C4	178.92 (7)	N2—C6—C7—C8	−178.21 (6)
C2—C3—C4—S1	0.05 (8)	N3—C7—C8—C9	−0.69 (11)
C1—S1—C4—C3	0.05 (6)	C6—C7—C8—C9	179.30 (6)
N2—N1—C5—C2	−178.20 (6)	C7—C8—C9—C10	0.02 (11)
C1—C2—C5—N1	−179.36 (6)	C8—C9—C10—C11	0.42 (12)
C3—C2—C5—N1	1.64 (11)	C7—N3—C11—C10	−0.38 (13)
N1—N2—C6—O1	3.95 (11)	C9—C10—C11—N3	−0.25 (13)
N1—N2—C6—C7	−175.76 (5)		

Symmetry codes: (i) −*x*+1, *y*−1/2, −*z*+1/2; (ii) *x*, −*y*+3/2, *z*+1/2.

### Hydrogen-bond geometry (Å, °)

**Table:**

*D*—H···*A*	*D*—H	H···*A*	*D*···*A*	*D*—H···*A*
N2—H2···O1^ii^	0.83 (1)	2.38 (1)	3.0717 (8)	140 (1)

Symmetry codes: (ii) *x*, −*y*+3/2, *z*+1/2.
